# Breast cancer in pregnancy: case report

**DOI:** 10.4314/pamj.v5i1.56195

**Published:** 2010-04-19

**Authors:** Momah Tobe, Carryl Stephen, Kondamudi Vasantha, Abraham Shirley, Rimpel Bernard, Xiao Phillip, Guevara Elizabeth

**Affiliations:** 1 The Brooklyn Hospital Center, Brooklyn, New York, NY 11201, USA

**Keywords:** Breast cancer, pregnancy

## Abstract

This case report is about a case of breast cancer in pregnancy at the Brooklyn hospital Center. Our patient`s case highlights some of the inherent causes of fatality in PABC and how to thread the line between the mother`s health and the baby`s safety to ensure a good outcome for both parties.

## Background

We report a case of breast cancer in pregnancy at our institution. The incidence of this disease is about 1.3 per 3,000 live births [1] and is the first reported case in our hospital over the last 15 years. In this case report we compare the standard of care received by our patient with currently recommended guidelines for managing Pregnancy Associated Breast Cancer (PABC) with special emphasis on under-30 year old pregnant females.

Frey’s syndrome can be socially debilitating and because of the difficulty in its management, preventive measures should be instituted during the initial surgery. To our knowledge, the longest latency of Frey’s syndrome after parotidectomy recorded in the literature is 50 years [1]. Our patient had parotidectomy at the age of 7 years and presented 40 years later with Frey’s syndrome.

## Patient and case report

A 27 year old female at ten weeks Gestational Age (G.A) presented with complaints of bloody nipple discharge and a palpable right breast mass of two weeks duration. There was gradual increase in the size of the right breast mass but no associated breast tenderness or skin changes. Patient had a past history significant for Chlamydia (1995), Cesarean Section (1996) and dilatation and Curettage for missed abortion in August 1999.

Patient had no history of cancer in the family and denied any use of alcohol, cigarettes or intra venous drugs. She worked for the Metropolitan Transport Authority and was actively involved at the World Trade Center rescue mission on September 11, 2001. She had her regular menstrual periods and denied ever using Oral Contraceptive Pills. Her menarche was at 11 years of age.

**Figure 1:**
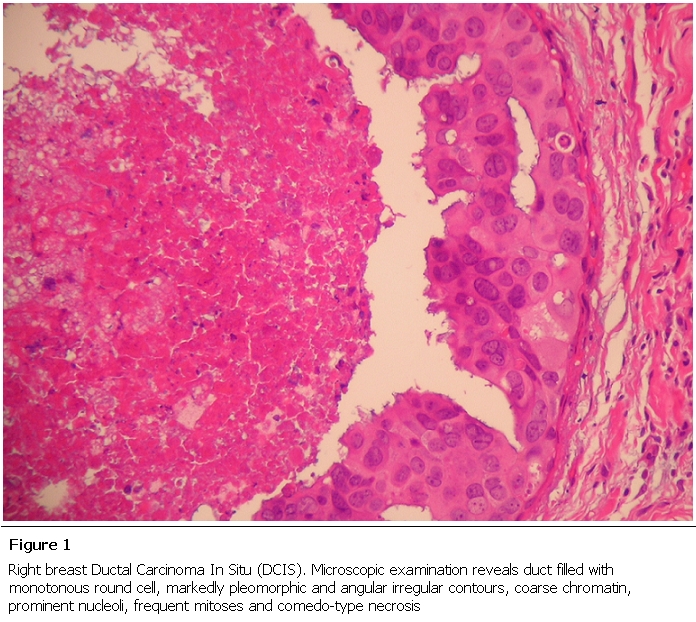
Right breast Ductal Carcinoma In Situ (DCIS). Microscopic examination reveals duct filled with monotonous round cell, markedly pleomorphic and angular irregular contours, coarse chromatin, prominent nucleoli, frequent mitoses and comedo-type necrosis.

On physical exam she had a Body Mass Index (BMI) of 34.1, a distended abdomen (consistent with gravid status) and a right breast mass that was firm and non-tender measuring 1.5 cm x 2 cm. On evaluation by breast ultrasound, a breast mass highly suspicious for breast malignancy was noted. After initial patient hesitancy, she (at twenty seven weeks of fetal gestational age) underwent a right breast incisional biopsy (under local anesthesia) with histopathology results showing estrogen receptor positive, progesterone receptor negative and human epidermal growth factor receptor 2 (HER2) positive invasive ductal Carcinoma with grade 3 multifocal Ductal Carcinoma In Situ (DCIS) (Figure 1).

**Figure 2:**
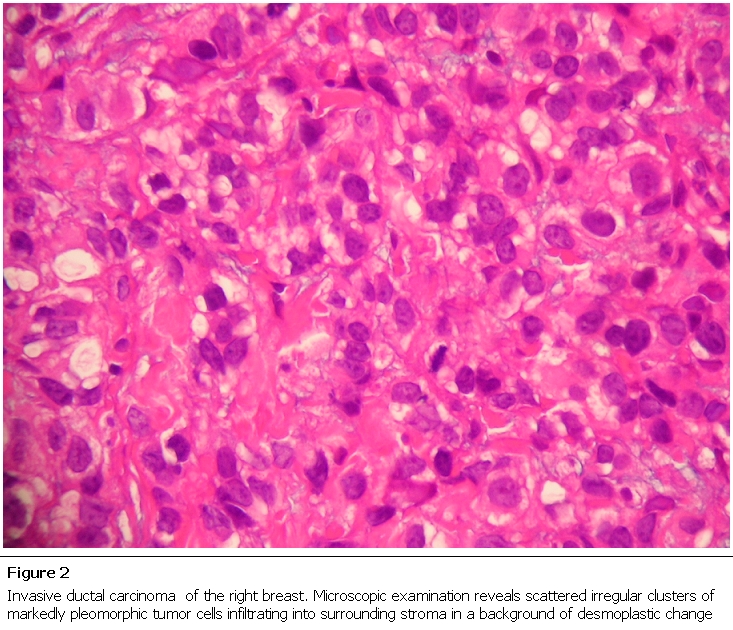
Invasive ductal carcinoma of the right breast. Microscopic examination reveals scattered irregular clusters of markedly pleomorphic tumor cells infiltrating into surrounding stroma in a background of desmoplastic change

At thirty two weeks G.A patient, under General Anesthesia, had a pre-term Cesarean-section followed by a right Modified Radical Mastectomy (MRM) and right axillary dissection. Surgery was uneventful with delivery of a 3.25 pounds male neonate and right breast mass (figure 2) positive for an 8 cm invasive ductal breast carcinoma with axillary lymph nodes involvement and consistent with a staging of T3N2M0. Two months after the mastectomy patient received the first of four cycles of chemotherapy (consisting of cytoxan and adriamycin), hormonal therapy (consisting of trastuzumab) for 12 months and subsequently began radiation therapy six months after the first dose of chemotherapy. During this period she formula fed her baby as initial breast feeding attempts from the left breast were unsuccessful.

Twelve months post-operatively patient underwent a transverse rectus abdominis myocutaneous (TRAM) flap reconstruction of the right breast. She had a complicated post-operative course with septicemia from abscesses in the TRAM flap site and eventually succumbed to hepatic encephalopathy secondary to liver and spine metastases a year and 7 months after her complicated cesarean delivery. No complications have so far been reported concerning the child born to our patient during this period.

## Discussion

When breast cancer is diagnosed in pregnancy both the patient and physician alike are faced with a difficult situation. The difficulty stems from how to preserve both mother and fetus without harm. Reports of an increased incidence of PABC [2] are due partly to the older ages (greater than 30 years) at which females are currently having children.

The most recent updates show that breast cancer is the second most common malignancy diagnosed during pregnancy[3] (after cervical cancer) and when diagnosed at ages 30 years or younger up to 10-20% are detected during pregnancy or within the first post-partum year [4]. A high index of suspicion is therefore warranted for a breast lump in an under thirty year old pregnant female and a breast ultrasound remains the gold standard for diagnoses. It may be difficult to palpate the mass due to increased breast engorgement and increased density of the breasts in pregnancy. Our patient`s young age combined with being pregnant a third time were considered protective in earlier studies [5] but a recent study by Albrektsen et al [6] showed that a risk (though less than the risk for above 30 year olds) exists in anyone having subsequent pregnancies [7] as their cases are characterized by a shorter time interval [8] to disease occurrence.

Our patient was managed surgically with post-partum MRM of the right breast and therapeutically by hormonal, chemo and radiation therapy. Current guidelines recommend surgical management similar to non-pregnant patients when discovered in the second or third trimesters with intra¬partum MRM and 2^nd^ or 3^rd^ trimester adjuvant chemotherapy [8]. Radiation therapy is contra-indicated in pregnancy as it is associated with mental retardation in the fetus. The delay of twenty-two weeks from suspicious breast mass to surgical excision and the subsequent six month delay from diagnosis to beginning radiation therapy contributed enormously to the eventual demise of our patient as the daily increased risk for developing metastasis for an untreated breast carcinoma in a pregnant women is 0.057% [9]. Even though patient hesitancy was partly responsible for delay in diagnosing and treating her in a timely manner, a delay in starting radiation therapy was in part secondary to her cesarean section (which delays radiation therapy compared to spontaneous vaginal delivery due to healing surgical incisions). It is worthy to note that our patient had a much faster tumor growth rate (300-400% increase in 4 to 5 months) and a much shorter survival period (T3N2MO breast cancer has a 5 year survival rate of 57%) than people with similar stages of breast cancer.

Our case is unique on the basis of the age our patient presented especially in the vicinity of no known family history. She refused to undergo the Breast receptor Cancer antigen (BRCA) I and II gene testing initially and attempts on other female family members were unsuccessful. The right breast re-construction started a downward spiral of her health secondary to multiple septic abscesses and combined with the spine and liver metastases only served to further undermine her already immune-compromised status. There are some reviews from the medical literature that criticize breast reconstructive surgery in a post partum PABC status post radiation therapy [10] patient but no consensus exists as regards to post-mastectomy reconstruction in patients such as ours.

## Conclusion

PABC is going to become an increasingly common feature in the African continent and will require medical providers increased awareness of guidelines for its management. Our patient`s case highlights some of the inherent causes of fatality in PABC and how to thread the line between the mother`s health and the baby`s safety to ensure a good outcome for both parties.

## Patient consent

Authors declared that they received written consent from the patient to publish this case report.

## Competing interests

The authors declared no conflicts of interests
